# Consensus–based approach to develop a measurement framework and identify a core set of indicators to track implementation and progress towards effective coverage of facility–based Kangaroo Mother Care

**DOI:** 10.7189/jogh.07.020801

**Published:** 2017-12

**Authors:** Tanya Guenther, Sarah Moxon, Bina Valsangkar, Greta Wetzel, Juan Ruiz, Kate Kerber, Hannah Blencowe, Queen Dube, Shashi N Vani, Donna Vivio, Hema Magge, Socorro De Leon–Mendoza, Janna Patterson, Goldy Mazia

**Affiliations:** 1 Save the Children, Washington DC, USA; 2 London School of Hygiene and Tropical Medicine, London, UK; 3 Kangaroo Mother Care Foundation, Bogota, Colombia; 4 Herbert Wertheim College of Medicine, FIU, Miami, Florida, USA; 5 Queen Elizabeth Central Hospital, Blantyre, Malawi; 6 Kangaroo Mother Care Foundation, Ahmedabad, India; 7 USAID, Washington DC, USA; 8 Partners in Health, Kigali, Rwanda; 9 Brigham and Women’s Hospital, Division of Global Health Equity, Boston, Massachusetts, USA; 10 Bless–Tetada Kangaroo Mother Care Foundation, Manila, Philippines; 11 Bill & Melinda Gates Foundation, Seattle WA, USA; 12 PATH, Washington DC, USA; 13 Maternal and Child Survival Programme, Washington DC, USA

## Abstract

**Background:**

As efforts to scale up the delivery of Kangaroo Mother Care (KMC) in facilities are increasing, a standardized approach to measure implementation and progress towards effective coverage is needed. Here, we describe a consensus–based approach to develop a measurement framework and identify a core set of indicators for monitoring facility–based KMC that would be feasible to measure within existing systems.

**Methods:**

The KMC measurement framework and core list of indicators were developed through: 1) scoping exercise to identify potential indicators through literature review and requests from researchers and program implementers; and 2) face–to–face consultations with KMC and measurement experts working at country and global levels to review candidate indicators and finalize selection and definitions.

**Results:**

The KMC measurement framework includes two main components: 1) service readiness, based on the WHO building blocks framework; and 2) service delivery action sequence covering identification, service initiation, continuation to discharge, and follow–up to graduation. Consensus was reached on 10 core indicators for KMC, which were organized according to the measurement framework. We identified 4 service readiness indicators, capturing national level policy for KMC, availability of KMC indicators in HMIS, costed operational plans for KMC and availability of KMC services at health facilities with inpatient maternity services. Six indicators were defined for service delivery, including weighing of babies at birth, identification of those ≤2000 g, initiation of facility–based KMC, monitoring the quality of KMC, status of babies at discharge from the facility and levels of follow–up (according to country–specific protocol).

**Conclusions:**

These core KMC indicators, identified with input from a wide range of global and country–level KMC and measurement experts, can aid efforts to strengthen monitoring systems and facilitate global tracking of KMC implementation. As data collection systems advance, we encourage program managers and evaluators to document their experiences using this framework to measure progress and allow indicator refinement, with the overall aim of working towards sustainable, country–led data systems.

An estimated 15 million babies are born prematurely each year, accounting for about 1 in 10 births worldwide [1]. Preterm birth, defined as birth before 37 completed weeks of gestation, is the leading direct cause of newborn mortality and morbidity [2,3]. Complications of prematurity are the primary cause of child death worldwide and also a risk factor for neonatal deaths from other causes, especially infections [3]. Globally, preterm birth complications contribute 3% of disability–adjusted life years (DALYs) for all ages and account for 38% of DALYs attributed to neonatal conditions [4]. The burden of mortality and morbidity due to preterm birth is heavily concentrated in south Asia and sub–Saharan Africa, where more than 60% of preterm births take place and health systems face multiple challenges to deliver high quality care [1,2,5].

The Every Newborn Action Plan (ENAP), a global multi–partner movement with the goal of ending preventable newborn deaths has set national targets of ≤12 neonatal deaths per 1000 live births by 2030 [6]. As the leading cause of newborn deaths, a focus on preterm birth and the associated complications are essential to achieving these ambitious goals. There are evidence–based, cost–effective interventions to prevent preterm birth and manage complications. As part of the evidence base for the ENAP, an epidemiological analysis estimated that up to 70% of preterm deaths could be averted through the provision of quality inpatient care [7]. Kangaroo Mother Care (KMC) is a critical part of inpatient care for preterm newborns, and also provides the foundation for improved outpatient and follow–up care of small babies [5,8]. In July 2015, the World Health Organization (WHO) released guidelines on interventions to improve preterm birth outcomes, which strongly recommend KMC for the routine care of neonates born weighing ≤2000 g as soon as they are clinically stable [9]. Birthweight is used as an indication for KMC initiation and a proxy for preterm birth given the challenges of accurate gestational age measurement in many low–resource settings.

Kangaroo Mother Care is defined by WHO as early, continuous and prolonged skin–to–skin contact between the mother (or other caregiver) and the baby, and exclusive breastfeeding (ideally) or feeding with expressed breastmilk [9]. Provision of KMC is embedded within a broader package of inpatient care for premature babies that involves supportive care (eg, infection prevention and management, respiratory support, etc.) and requires referral for higher level care when necessary and ongoing follow–up post–discharge [8]. In some more developed settings (eg, certain Latin American countries), KMC may be initiated at the facility and continued on an ambulatory basis with mothers returning to the facility frequently (as needed). Such an approach is only feasible in settings where health facilities are easily accessible and the appropriate infrastructure is in place. Studies show that continuous KMC implemented at health facilities can prevent up to 50% of deaths among babies ≤2000 g [10]. The practice of facility–based KMC also offers benefits beyond reduced mortality; compared with conventional neonatal care for small babies (incubator care), KMC reduces infections, hypothermia, and length of hospital stay and improves breast–feeding, weight gain and maternal–infant bonding [10]. Intermittent KMC, as tolerated, is increasingly being used for babies that are less stable to support clinical and developmental outcomes [9].

Despite the strong evidence base for KMC, progress in taking KMC implementation to scale has been slow [5,11,12]. While more than half of the 75 Countdown to 2015 countries report national policies recommending KMC, availability of KMC services is limited to a small number of central or teaching hospitals in all but a handful of countries ([13] and our unpublished results). A multi–country assessment of health systems bottlenecks to scale up of KMC in 12 African and Asian countries found that health financing, community ownership and partnership, health service delivery, leadership and governance and health workforce were perceived as major or significant barriers by nine or more countries [5]. One of the crosscutting challenges underpinning these barriers was effective information systems and data on KMC coverage and quality [5,12].

In an effort to accelerate and support the uptake of KMC, the Bill & Melinda Gates Foundation and partners released a call to action in 2013 for the global adoption of facility–based KMC and formed the KMC Acceleration Partnership (KAP) [11]. The call to action set an ambitious target of 50% coverage of KMC among preterm newborns by 2020 and emphasized the importance of measuring progress using robust metrics and indicators [11]. Similarly, to meet its ambitious goals, ENAP recognized the critical need for improved data on preterm, small and sick newborns to support the scale up of high impact interventions [6]. At the time of its launch in 2014, ENAP published a core set of indicators needed for tracking progress in reaching their goals [6]. Coverage of KMC is one of the core ENAP indicators, and also one of the indicators with some of the greatest identified data gaps [6,14]. At the time, there was no existing definition for a KMC coverage indicator. To achieve scale up of KMC, there is a need for consensus on a common set of indicators to track KMC implementation and progress to effective coverage. The ENAP metrics stream, therefore, prioritized work on defining and testing a measureable coverage indicator, but also emphasized the importance of developing process indicators to track content and quality. In conjunction with ENAP, the KAP initiated a consensus–based process to identify a core set of standardized indicators for KMC to facilitate country and global monitoring and evaluation of KMC efforts and inform the integration of data on KMC into national health management information systems (HMIS). Regular monitoring and reporting of these indicators will strengthen the global evidence base for KMC and inform approaches to strengthen scale–up of KMC [11,12]. Further, careful facility–level measurement of KMC service delivery is important for improving the quality of KMC services and can help avoid the phenomenon of “empty” scale–up.

The purpose of this paper is to describe the approach to develop a measurement framework and select and refine a set of indicators for monitoring implementation of facility–based KMC. The aim was to develop a focused list of indicators that would be relevant across settings and could be measured within existing health systems at scale. The challenges to establish an appropriate denominator for measuring coverage of KMC and options for testing are also discussed.

## METHODS

**Figure 1:**
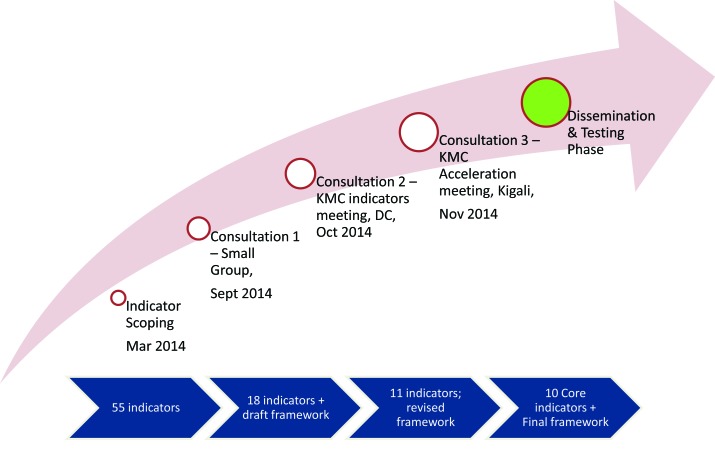
Overview of Kangaroo Mother Care (KMC) framework development and indicator selection process.

The KMC measurement framework and core list of indicators were developed through: 1) scoping exercise to identify potential indicators; and 2) face–to–face consultations with measurement and KMC experts to review candidate indicators and finalize selection (**Figure 1**).

### Scoping exercise to identify potential indicators

An initial list of candidate indicators was developed through a review of the grey literature (program documents and surveys) and consultations with KMC and measurement experts. We circulated a request for existing KMC indicators to members of the Newborn Indicators Technical Working Group (NITWG), an inter–agency working group convened by Saving Newborn Lives, and the KMC Acceleration Partnership. A total of 79 candidate indicators and data elements were extracted and summarized in an excel spreadsheet. This list was refined to 55 through sorting and removal of duplicates and organized by a standard results framework (impact, coverage, access, quality, demand, policy/enabling environment). The main sources of indicators in this initial list included the Fundacion Cangaru, Maternal and Child Health Integrated Program (MCHIP) KMC implementation Guide, various facility and household surveys conducted by programs implementing KMC (SNL Malawi Facility Assessment and household survey, Uganda Newborn Study (UNEST) survey, Ghana Newhints survey, Ethiopia household survey, South Africa Facility Assessment) and the Malawi HMIS.

### Consultations to review and finalize indicator selection

A series of face–to–face meetings were convened with KMC and measurement experts working at country and global level over a three month period. A full list of participants, their affiliations and area of expertise (measurement, KMC or both) are included in Table S1 in **Online Supplementary Document(Online Supplementary Document)**.

### Initial scoring and development of a measurement framework

A small group meeting with 12 members of the KAP and the NITWG was held September 5, 2014 to review and score the raw list of 55 indicators. A focused set of five criterion for initial scoring of the indicators was developed, which reflects commonly applied indicator selection criteria: feasibility (data can be collected with reasonable and affordable effort in low resource settings), reliability (data can be collected consistently over time), usefulness for decision–making (data are relevant and will help guide KMC programming), sensitivity (responsive to change), and specificity (focused on specific aspect, not overly broad) [15–17]. The group broke into smaller groups for in–depth discussion and scored each candidate indicator as high, medium, or low for each of the five criteria. The group recommended that a measurement framework specific to KMC should be developed to better organize the indicators and assist with prioritizing selection. Following the meeting, a core team representing the KAP, NITWG and ENAP metrics stream extracted the strongest indicators based on the scoring criteria for further development (preparation of full definitions, data source, and methods) and drafted a KMC measurement framework. The resulting 18 candidate indicators were then organized according to the draft framework.

### Refinement of framework and indicator list

In October, consultations were held with a broader group of newborn programmatic and measurement experts on October 6–7, 2014 in Washington DC. The 24 attendees, representing implementing agencies, donors and researchers, formed three small groups (national level/service readiness; facility–level/service delivery; and coverage) to review the KMC framework and each of the 18 candidate indicators in detail. Groups were provided with a series of questions for each indicator to guide their discussion and decision–making process. The group made recommendations about which indicators to retain, which to drop and areas for further research; further details on the discussion and outputs is available in the meeting report [18]. Following the meeting, the core team consolidated feedback and updated the measurement framework and refined the indicator list down to 11 candidate indicators. A smaller task team was delegated to work specifically on defining a feasible coverage indicator for KMC that could be tested as part of the ENAP metrics measurement improvement plan. This task team, alongside the core group, also undertook a preliminary mapping exercise to see what data were available, with a focus on assessing denominator options for generating a potential coverage indicator for KMC that could be tested as part of the ENAP measurement improvement plan.

### Finalization of framework and indicators

The final consultation took place on November 15, 2014 in Kigali, Rwanda as part of the KMC Acceleration meeting and focused on country level input. Eighteen participants, including individuals supporting KMC implementation in nine countries (Bangladesh, Malawi, Nigeria, Rwanda, India, Indonesia, Philippines, Uganda and South Africa) gave feedback on the measurement framework and reviewed each candidate indicator to assess availability, feasibility and usefulness considering their country context. Participants were split into two groups. One group worked specifically on the coverage indicator, and the other group focused on readiness indicators and facility level data for tracking service delivery and quality of care. In each group, a presentation was made to provide an overview of progress to date, review each indicator in detail and identify priority areas for discussion. Participants in the service readiness and facility data group were asked to use post–it notes to record information on availability/data source, data users, collection methods, and challenges for each indicator in their setting and then vote whether the indicator was ready to go, needed more work/unsure or should be dropped. Participants in the coverage group, reviewed the work carried out by the ENAP metrics KMC task team (**Box 1**) and discussed a feasible a measurable coverage indicator. In view of the challenges in measuring a denominator, the group reached consensus through placing individual votes between use of <2500 g, total facility births or estimated live births. Based on the feedback, the core team finalized the framework and list of indicators.

Box 1 **The challenge of measuring KMC coverage**

**What is coverage measurement and why is it challenging for KMC?**

A coverage indicator aims to measure the number of individuals that receive a specific intervention or treatment within a given population in need of the intervention. The numerator is measured as the total number of individuals that received the intervention and the denominator is the total population, usually those that could have benefitted from that specific intervention or treatment. For KMC, neither the numerator nor the denominator are easy to define or measure. KMC is not a one–off contact with the health system; many of the components of KMC are processes (eg, continuous skin–to–skin contact, follow up care). And measurement of specific interventions is a challenge when only by a small group or sub population benefit from that intervention. Defining whether or not an infant could benefit from KMC requires a level of clinical judgement and more precise metrics than those reported by most routine information systems in LMIC.

**The ENAP metrics KMC task team**

ENAP metrics assembled a KMC task team with experts in measurement and programme implementation drawing on expertise from the KMC acceleration partnership and wider groups. Different numerators and denominators were proposed and discussed based on their definition and the feasibility of measurement.

**Numerator challenges**

The evidence base for mortality impact of KMC is currently for infants weighing 2000 g or less. However, in some low and middle income countries where programmes have been extended, eligibility criteria for entry to KMC may be for babies up to 2500 g. Coverage of most maternal and newborn interventions in many settings is still measured through household surveys and relies on maternal recall up to five years after the birth in question. Even though mothers can accurately recall KMC, even years after the event, the sample size needed to gather representative data through a household survey may be prohibitively large^15^. Typically, facility based assessments capture information on infrastructure, processes and service readiness, and are best suited to measure the number of facilities that are prepared to provide components of the service (eg, sufficient trained staff, space, and equipment). In most settings, the number of newborns initiated on facility–based KMC is measured either through hospital admission or care records, but currently these data are rarely reported into national health information systems.

**Denominator challenges**

The denominator was the most technically challenging and a list of options were proposed. A large proportion of newborns do not have their weight recorded at birth and even where birthweight is recorded, there is a known tendency for “heaping” of data, especially at measures of 2500 g and 2000 g. Given the difficulty in accurately capturing all those babies in need of KMC, especially through existing data collection systems, using total live births as the denominator to give a proxy was considered. This has been done with other interventions where the aim is not for 100% coverage, such as C–section, to generate a rate that is benchmarked against a target threshold. Recent estimates suggest a variation in preterm birth rates of between 4–18% of total lives births in different countries. This means that the KMC rate in each country may indicate a different unmet need and target thresholds would need to vary between settings to reflect these differences as well as variation in numbers of full–term LBW and pre–term babies. As an important limitation, if total live births is used as a denominator, it does not reflect whether the babies that received it were drawn from the population that could have benefitted from KMC.

**Proposed indicator**

The ENAP KMC task team established that it is not possible to capture all of the components of KMC in one coverage indicator as many of these refer to processes that happen over a period of time. Household surveys are unlikely to be a feasible approach to measure KMC coverage and increasingly, health facility assessments are starting to measure key components of KMC care. Of all the available options, **the number of newborns initiated on facility based KMC** gives a representation of the number of newborns initiating the care. Task teams agreed the indicators would need rigorous testing for validity and feasibility with a variety of different denominator options including, **live births in the facility, estimated live births and eventually target population for coverage (total number of newborns ≤2000 g).**

**Figure 4:**
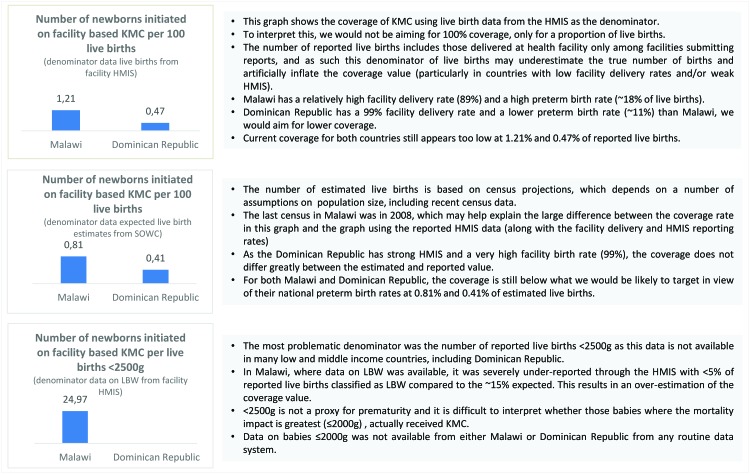
Analysis of denominator options for Kangaroo Mother Care (KMC) coverage indicator in Malawi and Dominican Republic.

As a preliminary exercise, the task team approached a select few LMIC countries for data on the KMC numerator, which is available through a limited number of HMIS and many hospital registration systems. To demonstrate the numerator with different denominator options, task teams present three graphs showing the proposed numerator over total reported live births, total reported live births <2500 g and estimated live births for two countries, Malawi and Dominican Republic (**Figure 4**).

**What are the next steps?**

As national facility based data and health information systems become more advanced, the ideal is to develop more precise indicators, but these are not currently available in most of the countries where the unmet need for KMC is arguably the greatest and there are the most data gaps. It is critical to improve the recording and reporting of birth weight in facilities. Given the importance of prematurity as a direct cause of death and as a risk factor for morbidities and death from other causes (eg, infections), developing simplified tools for measuring gestational age is critical to plan for programmes, to improve the evidence base and to develop more precise indicators of unmet need. If such data were available in more settings, indicators based on specific weight or gestational age criteria could be measured. Existing data sets from countries with established KMC programmes and accurate assessment of gestational age and birthweight could be used for testing the denominators and proposed numerators. The ENAP metrics measurement improvement plan has a five year plan set out to test the validity and feasibility of a number of numerator and denominator options for all the ENAP core indicators with the objective of institutionalizing a KMC coverage indicator in global accountability mechanisms by 2020.

## RESULTS

### KMC measurement framework

**Figure 2:**
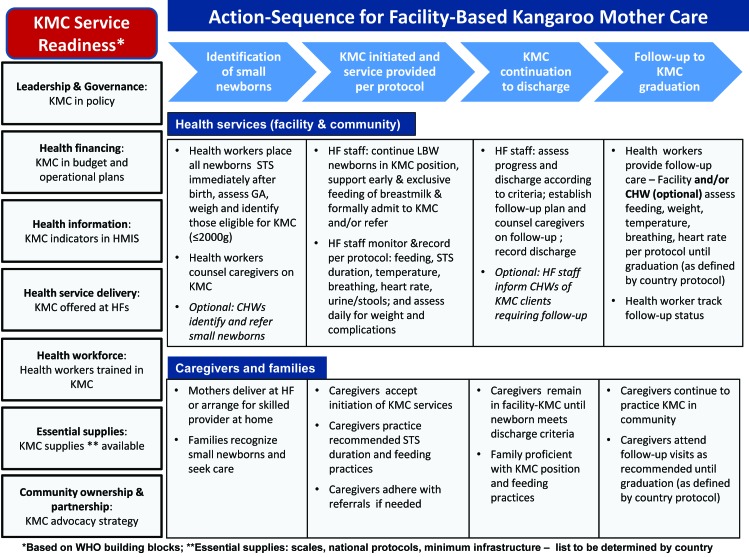
Measurement framework for Kangaroo Mother Care (KMC).

**Figure 2** shows the KMC measurement framework, which was developed to guide the identification and prioritization of core indicators. The framework includes two main components: 1) KMC service readiness and 2) action sequence of KMC service delivery. The seven service readiness elements are based on the WHO building blocks framework, and specify what minimum elements should be in place to support national–level implementation of KMC [19]. The action sequence of service delivery outlines four main steps necessary for provision of KMC at health facilities: identification of small babies; KMC initiation per protocol; KMC continuation to discharge; and follow–up to KMC graduation. Essential actions for health service providers and for caregivers and families are outlined in broad terms for each step in the action sequence.

### KMC core indicators

**Figure 3:**
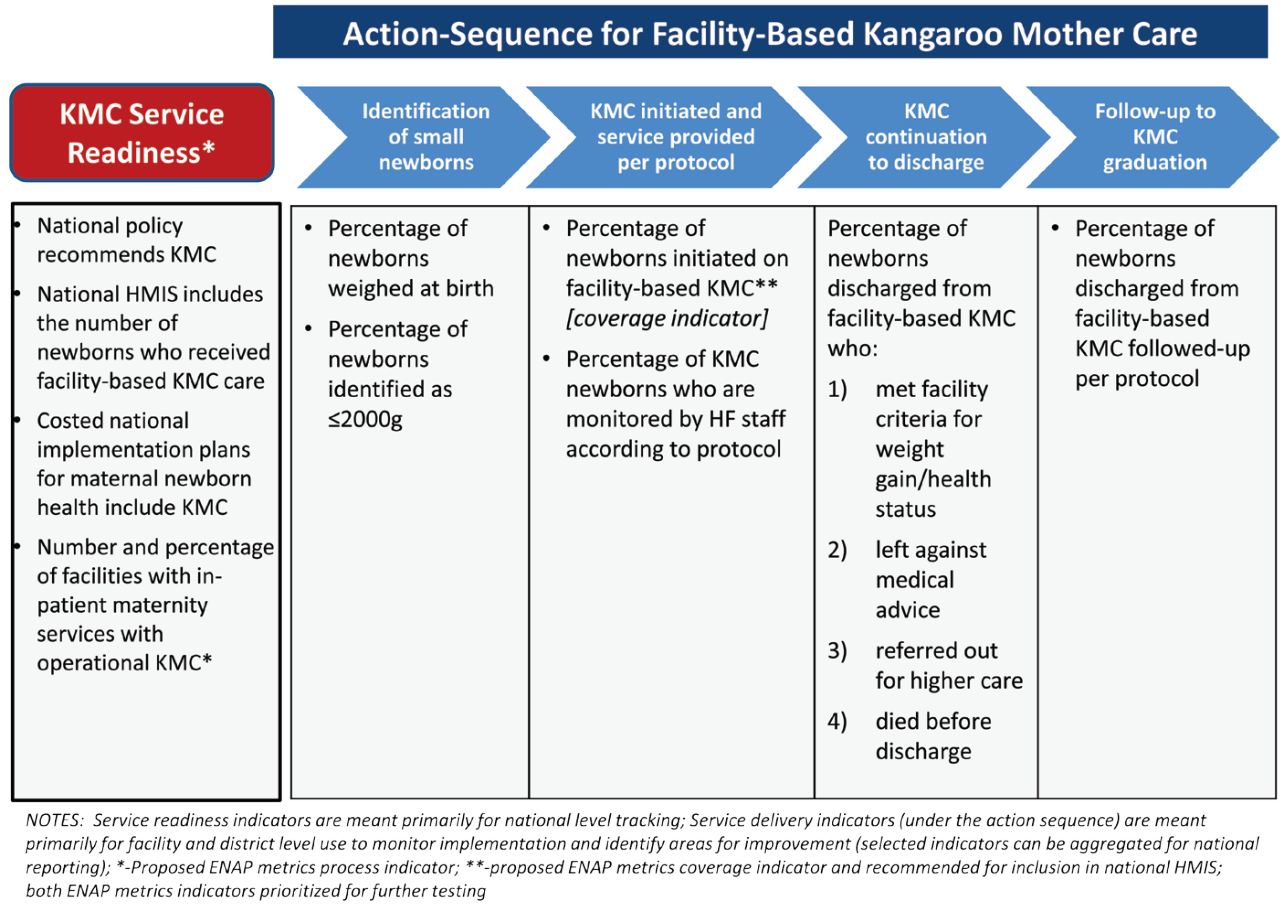
Summary of core Kangaroo Mother Care (KMC) indicators according to framework.

The 10 KMC core indicators are summarized in **Figure 3** according to the framework and defined in **Table 1**. Table S2 in **Online Supplementary Document(Online Supplementary Document)** provides further information on the limitations and additional data collection considerations for each indicator.

**Table 1:** KMC indicator definitions and data sources

Indicator	Metric	Main purpose	Data source(s) and methods of collection	Frequency
**KMC in national policy:** National policy recommends KMC	**Yes** = national policy recommends KMC	National reporting/monitoring	National policy documents – record review; Key informants through interview	Annually or less
	**No** = national policy does not recommend KMC			
**KMC indicator in HMIS:** National HMIS includes the number of newborns who received facility–based KMC care	**Yes** = national HMIS includes the number of newborns who received facility–based KMC	National reporting/monitoring	HMIS documents – record review; Key informants through interview	Annually or less
	**No** = national HMIS does not include the number of newborns who received facility–based KMC			
**Costed plan includes KMC:** Costed national implementation plans for maternal newborn health include KMC	**Yes** = costed plan or plans to scale up maternal, newborn and child health intervention includes KMC components	National reporting/monitoring	Costed plans – record review; Key informants through interview	Annually or less
	**No** = no costed implementation plan OR costed implementation plan does not include KMC components			
**KMC service availability:** Percentage of facilities with in–patient maternity services with operational KMC	Numerator: Number of health facilities in which KMC is operational*	National reporting/monitoring	Facility assessments and MOH records (collected through supervision or periodic audits)	Annually or less
	Denominator: Number of health facilities with inpatient maternity services			
**Weighed at birth:** Percentage of newborns weighed at birth	Numerator: Number of newborns weighed at birth†	Facility monitoring	Interviews with mothers + child health card review – collected through household surveys; L&D registers – collected through record review as part of facility assessment or supervision	Periodic for household surveys; routinely (monthly/quarterly) depending on need
	Denominator: Number of live births			
**Identification of newborns ≤2000 g:** Percentage of live births identified as ≤2000 g	Numerator: Number of newborns identified as ≤2000 g	Facility monitoring	L&D registers – collected through HMIS (see notes) or through register review as part of supervision or facility assessment	Routinely (monthly/quarterly)
	Denominator: Number of live births			
**KMC coverage**‡**:** Percentage of newborns initiated on facility–based KMC	Numerator: Number of newborns initiated on facility–based KMC§ Denominator: Expected number of live births OR expected number of LBW babies	Facility monitoring & National reporting/monitoring	KMC registers – reported through HMIS or collected through register review as part of facility assessment; Denominator available through national and global estimates updated annually	Annually
**KMC monitoring:** Percentage of KMC newborns who are monitored by health facility staff according to protocol	Numerator: Number of newborns admitted to KMC who are monitored by health facility staff according to protocol (includes at minimum: assessing feeding, STS duration, weight, temperature, breathing, heart rate, urine/stools)	Facility monitoring	KMC patient charts – collected through record review as part of facility assessment/supervision visits	Quarterly or less; to be determined at country level
	Denominator: Number of newborns initiated on facility–based KMC			
**Status at discharge from KMC facility:** Percentage of newborns discharged from KMC facility who: met facility criteria for weight gain/health status; left against medical advice; referred out; or died before discharge	Numerator: Number of newborns discharged from facility–based KMC who: 1) met facility criteria for weight gain, health status, feeding, thermal regulation, family competency, etc; 2) left against medical advice; 3) referred out for higher level care; 4) died before discharge	Facility monitoring	KMC registers – reported through HMIS or collected through register review as part of facility assessment	Routinely (monthly/quarterly)
	Denominator: Number of newborns discharged from facility–based KMC			
**KMC follow–up:** Percentage of newborns discharged from facility–based KMC that received follow–up per protocol	Numerator: Number of newborns discharged from facility–based KMC that received follow–up per protocol	Facility monitoring	KMC registers/records – reported through HMIS or collected through register review as part of facility assessment and/or) Interviews with caregivers/mothers of newborns discharged from KMC	Routinely (monthly/quarterly)
	Denominator: Number of newborns discharged alive who received facility–based KMC¶			

KMC – Kangaroo Mother Care; HMIS – Health Management Information System; MOH – Ministry of Health; LBW – low birth weight; L&D – Labour and Delivery; STS – skin–to–skin

*KMC elements already collected through Service Provision Assessments (SPA) include: staff receiving in-service training on KMC; identified space for KMC; and availability of functional infant scale. This indicator has been prioritized for further testing by the KMC Acceleration and ENAP metrics group, with particular focus on identifying and testing additional KMC elements for inclusion in future harmonized facility assessments, supervision checklists and MOH audits.

†Countries will need to define a timeframe for ‘weighed at birth’. In some settings, this may include babies weighed at admission to the health care facility within a certain timeframe after delivery and babies weighed at home by a trained provider with weight documented on a maternal held record.

‡Indicator recommended as priority for inclusion in national HMIS.

§This may include facility–initiated ambulatory KMC as in Latin America (eg, Colombia); this indicator has been prioritized for further testing by the KMC Acceleration and ENAP metrics group, with particular focus on establishing the most feasible, valid, and reliable denominator and benchmarks for interpretation.

¶Countries should define their own denominator based on the national protocol for follow–up care of small and sick newborns, with an ideal denominator that captures all those infants discharged alive that were potential candidates for KMC.

### Service readiness indicators

Consensus was reached on four indicators of service readiness, namely national level policy for KMC, availability of KMC indicators in HMIS, costed operational plans for KMC and availability of KMC services meeting national minimum standards at facilities with inpatient maternity services. The service readiness indicators are qualitative milestones or benchmark indicators, which build on existing indicators and data collection efforts at national/global level, such as the Countdown to 2015 policy indicators. Primary data sources are Ministry of Health and implementing partners and the data can be aggregated at global level to track progress across countries. Availability of data are mixed. The indicator on national level policy for KMC was added in 2014 to the set of policy indicators tracked in Countdown to 2015 and data are available for two years (2014 and 2015), but there are no clear plans for tracking post 2015 [14]. The other three indicators are not currently tracked and would need to be collected in coordination with countries. Limited data on the availability of KMC services at health facilities are currently captured through several large facility assessment tools, including the Demographic and Health Survey Program’s (ICF International) Service Provision Assessment (SPA) and the WHO’s Service Availability and Readiness Assessment (SARA) and the revised Emergency Obstetrics and Newborn Care (EmONC) assessment tools [20]. However, these assessments are conducted infrequently, and countries investing in scaling up KMC services may need to establish other more frequent mechanisms to track KMC service availability, such as supervision or periodic audits. Further work is also needed to define ‘operational’ KMC, but at minimum it should specify availability of trained staff, space, and supplies. Defining these minimum components was also considered critical to the ENAP metrics measurement improvement plan, which recommended tracking the availability of KMC services as a process indicator [14].

### Service delivery indicators

Six indicators were identified for service delivery, including weighing of babies at birth, identification of those ≤2000 g, initiation of facility–based KMC (coverage indicator), monitoring of KMC for quality, status of babies at discharge from KMC and level of follow–up according to national protocol. The service delivery indicators focus on capturing service utilization and elements of quality (both process and outcomes) and are intended for use primarily at the facility and district level for assessing KMC implementation and identifying program improvement needs. As such, the primary data sources are routine facility records and the list of recommended indicators was kept as short as possible to minimize burden on health staff.

Two indicators focus on identification of babies eligible for KMC. The percentage of newborns weighed at birth is captured by both the Demographic Health Survey (DHS) and the Multiple Indicator Cluster Survey (MICS), but only every few years. Weighing of babies at birth can also be estimated on a more routine basis using facility labour and delivery (L&D) records, although in most cases only facility births would be included and quality of recording is often poor. The DHS and MICS household surveys also estimate the percentage of low birth–weight babies, defined as birth weight <2500 g. However, as the evidence base for KMC is for babies weighing ≤2000 g at birth, the recommended indicator reinforces this cut–off and encourages routine data collection through facility L&D records. Most countries have space to record actual birth weight, but to our knowledge very few high burden countries currently collate and report on the number of live births weighing ≤2000 g. In addition to improving measurement of birth weight, investment in approaches to strengthen assessment of gestational age during antenatal care and at delivery are necessary to better target KMC interventions towards who would benefit most as the proportion of LBW babies that are pre–term will vary by setting.

The percentage of babies initiated on facility–based KMC was identified as a coverage indicator. Defining the denominator for a KMC coverage indicator proved especially challenging. The ideal denominator would be the number of babies born weighing less or equal to 2000 g. Yet as noted earlier, few if any low–income countries currently reliably capture such data for all births. Even for those births occurring in facilities, weight is not always recorded and if recorded, not always accurate and reliable. In the interim, several denominators are recommended for further testing. The preliminary mapping exercise suggested that using the expected number of live births may be the preferred denominator until measurement of birth weight improves (see **Box 1**). In this scenario, a benchmark value or range would need to be established for interpretation, similar to that used for C–section rates.

Three of the recommended service delivery indicators serve as proxies for quality of care processes and outcomes. While in facility, KMC babies require daily monitoring to assess and record their positioning, feeding, and weight gain and to check for signs of illness or other complications. One core indicator tracks the percentage of KMC babies who are monitored according to the national protocol by reviewing patient charts or other relevant facility records, through supervision visits or periodic assessments (monitoring adherence with recommended processes). The status of babies at the time of discharge from KMC is also an important proxy measure of quality of care, monitoring overall performance through a critical outcome indicator. Status at discharge should be captured in a KMC or postnatal register and include the following categories: met facility criteria for weight gain, health status, feeding, thermal regulation, and family competency with KMC (the ideal); died before discharge; left against medical advice (defaulters); and referred out for higher level care. These categories are similar to those used for community management of acute malnutrition (CMAM) programs for performance monitoring; however unlike CMAM, protocols differ substantially by country and there is insufficient data and experience to establish international minimum performance standards for KMC [21]. The third proxy indicator for quality relates to the level of follow–up post–discharge from facility KMC, which can be regarded as both a process and intermediate outcome. It is common for low birth weight and preterm babies to be discharged at 1500–1800 g to reduce exposure to nosocomial infections and allow space for other patients. Adherence to regular follow–up care that involves tracking growth and addressing other complications of prematurity is critical for improved outcomes of these still vulnerable preterm babies. Improving measurement of gestational age during pregnancy and/or by clinical assessment of the baby is essential for better targeting of clinical interventions and identification of infants who will require long–term and specialized care and follow–up. As discharge criteria and follow–up schedules vary by country, the indicator definition for follow–up will need to be tailored in each country accordingly. Assessing follow–up through routine sources can be complicated if babies receive follow–up care at different facility than where they received KMC, in which case periodic assessments may be required to supplement routine data.

## DISCUSSION

To the best of our knowledge, this is the first attempt to use a global consultation process to identify a prioritized set of core indicators to track country progress towards scaling up KMC. Both ENAP and the KMC Acceleration Partnership have set ambitious goals for reducing newborn morbidity and mortality through improved coverage of high impact interventions, including KMC [6,11]. Better quality data and measurement of KMC will be critical in accelerating progress of implementation and supporting scale up of the intervention. As has been seen with child health programs (eg, vaccinations) good quality, comparable data allows for informed planning, decision–making and targeting of programs. As direct complications of prematurity are now the leading cause of child death, comparable data are critical to foster global visibility, policy attention and accountability structures within the Sustainable Development Goals for child health. This requires a consistent approach to measurement of KMC with standardized indicators and data collection methodologies that can be captured in sustainable, country–driven health information systems. We employed a consensus–based process to develop a measurement framework and identify a set of 10 core indicators for measuring progress of KMC implementation. The resulting framework can be used to help program managers at the country level plan and set milestones that will be comparable between different settings. At a facility level, program implementers can use the service delivery indicators to identify areas for quality improvement.

The indicator selection and refinement process had several strengths. We engaged a broad range of KMC and measurement experts representing global and country level perspectives and diverse technical and methodological expertise. The candidate indicators were selected through a literature review, including peer reviewed and grey literature (surveys and program documents), and canvasing of KMC researchers and implementers to ground the work in experience with existing measures. We conducted a preliminary mapping exercise to look at availability of data for some of the most critical indicators and we consulted with country–based KMC implementers to assess the feasibility and relevance of the proposed indicators. To avoid overburdening health systems and frontline workers with unnecessary data collection requirements, we intentionally kept the list to a minimum set and focused on indicators with potential to be collected within existing, sustainable systems. Finally, the overlap between members of the ENAP metrics stream and the KMC Acceleration Partnership facilitated close collaboration and alignment of the recommended process and coverage indicators. This collaboration also allowed for wide consultation and shared learning with other groups facing similar challenges to measure interventions for newborns requiring extra care (eg, neonatal resuscitation, treatment of neonatal infections) [14].

The development of a measurement framework specific to KMC played a critical role in guiding the process of selecting and refining the indicators. Inclusion of the WHO building blocks helped ensure a health systems approach and the action sequence identified the major steps that need to take place to deliver high quality KMC services. Use of the framework allowed us to ensure indicators were evenly spread along the continuum of care from service readiness to service delivery. The framework also provides a useful reference point for program implementers, evaluators and researchers to identify additional indicators on aspects of readiness and service delivery. The intent was not to create a rigid framework, but to prioritize indicators that are relevant to implementation across a wide range of settings. The expectation is that individual programs will identify additional indicators that are program specific and adapt the framework to fit their context and data collection capacity.

As mentioned in the results, data availability is limited for most of the recommended KMC indicators. For countries in early stages of introducing KMC, the focus should be on tracking progress against the service readiness indicators. Once KMC is integrated within packages of care for preterm babies, countries can design and test data collection systems to capture more of the service delivery indicators. For countries with more established KMC services, efforts should be made to review their existing health information systems to determine the best way to integrate the recommended indicators such that select indicators are reported up to the national level. While nearly all service delivery indicators could be aggregated and reported through a national HMIS, given system constraints in most settings, priority should be given to capturing KMC initiation for tracking coverage. Countries will need to tailor some of the indicator definitions, particularly for KMC service availability, KMC monitoring, and KMC follow–up, to align with national protocols and clinical guidelines.

The core list of KMC indicators should be considered in light of the limitations of the process and of the indicators themselves. First, due to time constraints we were unable to undertake a formal systematic review of the literature and may have missed some relevant information. However, we did reach out to renowned KMC experts to share their experiences and materials and the majority of candidate indicators were extracted from these grey sources. Second, while we convened a series of consultations with a wide range of experts including representatives directly involved in country implementation, we were not able to sufficiently involve the principal end–users of the service delivery indicators – namely managers and service providers at district and facility levels. Third, given the aim was to develop a focused list of indicators suitable for routine systems, several aspects central to quality implementation of KMC are not reflected in the set of core indicators. Approaches to capture aspects such as timely initiation of KMC, extent of skin–to–skin and feeding practices, referral completion and health outcomes were discussed in–depth and considered only feasible within the context of research settings or special studies for the time being. In 2016, the WHO released a set of standards for improving quality of maternal and newborn care in health facilities and recommended two indicators for facilities to use to evaluate quality of KMC care; these draw attention to and have the potential to reinforce facility–level quality improvement efforts. However, measuring these quality of care indicators would require detailed information captured through daily patient charts and may not be feasible for routine monitoring and national aggregation in most settings [22].

Future work will include developing guidance for the indicators such as detailed reference sheets outlining how to collect and use the data effectively and supporting country–level partners to adapt and use the indicators. Both the KAP and ENAP metrics offer platforms for disseminating such materials to a wide audience and to collate and share additional resources and experiences gathered through collecting the indicators. The KAP regional communities of practice in Africa and Asia will convene meetings in 2016 and 2017 and provide an important opportunity to engage country–level partners to further refine the indicators. A critical next step is initiating special studies to test and validate the recommended KMC coverage indicators as outlined in the ENAP measurement improvement roadmap (see **Box 1**) [14]. This will be embedded in work to test all of the core ENAP coverage indicators for newborns with complications requiring extra care (antenatal corticosteroids, neonatal resuscitation and treatment of neonatal infection) that face similar measurement challenges. Data collection is under way to test a range of numerator and denominator options for validity (eg, sensitivity and specificity of the indicators), feasibility of measurement and usefulness through country hubs in Bangladesh and Tanzania. Another important area of future work is to establish a coordinated mechanism for global tracking of a sub–set of the core indicators to assess progress towards the KAP and ENAP goals. This will require harmonized investments in strengthening country health information systems, prioritizing capture of data to generate coverage estimates following validation efforts, as well as a system for global reporting.

## CONCLUSIONS

As KMC accelerates globally, a standardized approach to measuring implementation and progress towards effective coverage is needed. The indicators presented in this paper, identified with input from a wide range of global and country–level KMC and measurement experts, can aid efforts to strengthen monitoring systems and facilitate global tracking of KMC implementation. As data collection systems advance, we encourage program managers and evaluators to document their experiences using this framework to inform further progress and indicator refinement with the overall aim of working towards sustainable, country–led data systems.
