# Supplementary material for: Consensus–based approach to develop a measurement framework and identify a core set of indicators to track implementation and progress towards effective coverage of facility–based Kangaroo Mother Care
Source: J Glob Health. 2017 Oct 10;7(2):020801. doi: 10.7189/jogh.07.020801 (PMC5644361; doi:10.7189/jogh.07.020801)
Supplement: Online Supplementary Document [file jogh-07-020801-s001.pdf]

Online Supplementary Document

Guenther et al. Consensus–based approach to develop a measurement framework and identify a core set of indicators to track implementation and progress towards effective coverage of facility–based Kangaroo Mother Care

J Glob Health 2017;7:020801

Table S1: List of participants by consultation

Notes: *Bolded names represent members of the core indicator development team; * - denotes measurement expertise; ‡ denotes KMC expertise; and € denotes member of ENAP metrics task team for KMC*

**Initial scoring and development of measurement framework – September 5, 2014, Washington DC, USA**	
Hannah Blencowe*‡€<br>*London School of Hygiene and Tropical Medicine* (LSHTM), London, UK	Jennifer Callaghan*<br>*John's Hopkins University (JHU)*<br>Baltimore, USA
Mary Drake*<br>*Maternal Child Support Program (MCSP)*<br>Washington, DC, USA	**Tanya Guenther*€**<br>*Saving Newborn Lives (SNL), Save the Children*<br>Washington, DC,USA
Neena Khadka‡<br>*MCSP/Save the Children,* Washington, DC, USA	Goldy Mazia*‡€<br>*PATH/MCSP*, Washington, DC, USA
**Sarah Moxon*‡€**<br>*LSHTM/Every Newborn Action Plan (ENAP) Metrics*, London, UK	Magdalena Serpa‡<br>*PATH/ MCSP,* Washington, DC, USA
**Bina Valsangkar‡€**<br>*SNL, Save the Children,* Washington, DC, USA	Lara Vaz*<br>*SNL, Save the Children,* Washington, DC,USA
Brendan Wackenreuter*<br>*SNL, Save the Children,* Washington, DC, USA	**Greta Wetzel***<br>*SNL, Save the Children,* Washington, DC, USA
**Refinement of framework and indicators, KAP meeting, Oct 6-7, 2014, Washington DC, USA**	
Stella Abwao‡<br>*Save the Children,* Washington, DC, USA	Asri Adisamita‡<br>*Universitas Indonesia*, Jakarta, Indonesia
Hannah Blencowe*‡<br>*LSHTM*, London, UK	Grace Chan*‡<br>*Harvard University*, Boston, USA
Queen Dube‡<br>*Queen Elizabeth Central Hospital,* Blantyre, Malawi	Cyril Engmann‡<br>*PATH*, Seattle, Washington, USA
John Grove*<br>*Bill and Melinda Gates Foundation*, Seattle, Washington, USA	Tanya Guenther*€<br>*SNL, Save the Children,* Washington, DC,USA
Zelee Hill*<br>*University College London*, London, UK	Steve Hodgins*<br>*SNL, Save the Children,* Washington, DC,USA
Lily Kak‡<br>*USAID*, Washington, DC, USA	Kate Kerber*‡€<br>*SNL, Save the Children,* Edmonton, Alberta, Canada
Sarah Moxon*‡€	Winnie Mwebesa‡

LSHTM/ENAP metrics, London, UK	*Save the Children,* Washington, DC, USA
Harriet Nambuya‡<br>*Save the Children Uganda*, Kampala, Uganda	Juan Ruiz*‡€<br>*Kangaroo Mother Care Foundation*, Bogota, Colombia
Magdalena Serpa‡<br>*PATH/ MCSP,* Washington, DC, USA	Bina Valsangkar‡€<br>*SNL, Save the Children,* Washington, DC, USA
Shashi N. Vani‡<br>*Kangaroo Mother Care Foundation*, India	Lara Vaz*<br>*SNL, Save the Children,* Washington, DC,USA
Donna Vivio‡<br>*USAID*, Washington, DC, USA	Brendan Wackenreuter*<br>*SNL, Save the Children,* Washington, DC, USA
Steve Wall‡<br>*SNL, Save the Children,* Washington, DC, USA	Greta Wetzel*<br>*SNL, Save the Children,* Washington, DC, USA

Finalization of framework and indicators, KAP meeting, November 15, 2014; Kigali, Rwanda

Service Readiness Indicator Group:

Queen Dube‡<br>*Queen Elizabeth Central Hospital,* Blantyre, Malawi	Ruth Davidge‡<br>*Neonatal Nurses Association of South Africa (NNASA),* Kwa-Zulu Natal, South Africa
Tanya Guenther*€<br>*SNL, Save the Children,* Washington, DC,USA	Erin Hunter*<br>*Johns Hopkins University*, Dhaka, Bangladesh
Hema Magge*‡<br>*Partners in Health*, Kigali, Rwanda	Socorro de Leon Mendoza‡<br>*Bless-Tetada KMC Foundation,* Philippines
Silvia Nurdin‡<br>*Save the Children Indonesia*, Jakata, Indonesia	Shashi N. Vani‡<br>*Kangaroo Mother Care Foundation*, India
Donna Vivio‡<br>*USAID*, Washington, DC, USA	Greta Wetzel*<br>*SNL, Save the Children,* Washington, DC, USA

KMC Coverage Indicator Group:

Nathalie Charpak‡<br>*Kangaroo Mother Care Foundation*, Bogota, Colombia	Eni Gustina‡<br>*Ministry of Health Indonesia*, Jakarta, Indonesia
Neena Khadka‡<br>*MCSP/Save the Children*, Washington, DC, USA	Goldy Mazia*‡<br>*PATH/MCSP*, Washington, DC, USA
Suleiman Mohammed‡<br>*Federal Medical Centre Nigeria*, Nigeria	Sarah Moxon*‡€<br>*LSHTM/ENAP metrics*, London, UK
Evelyn Zimba‡<br>*USAID*, Lilongwe, Malawi	Peter Waisawa*<br>*Makerere University School of Public Health*, Kampala, Uganda

Table S2. KMC indicator limitations and additional data collection considerations

Indicator	Metric	Data source(s) and methods of collection	Limitations/challenges	Data collection considerations and possible alternatives
**KMC in national policy:** National policy recommends KMC	**Yes** = national policy recommends KMC<br>**No** = national policy does not recommend KMC	National policy documents - record review; Key informants through interview	Indicator does not capture whether the policy is aligned with WHO recommendations for KMC. Policy may not reflect service availability.	Key informants may not be reliable, where possible, 'yes' values should be supported by review of policy documents
**KMC indicator in HMIS:** National HMIS includes the number of newborns who received facility-based KMC care	**Yes** = national HMIS includes the number of newborns who received facility-based KMC<br>**No** = national HMIS does not include the number of newborns who received facility-based KMC	HMIS documents - record review; Key informants through interview	Indicator does not assess data completeness, quality, timeliness or accuracy, nor whether the data are used for program decision-making	HMIS documents should be used to verify 'yes' values. Ideally, should be supplemented with periodic data quality audits to better assess data availability and quality.
**Costed plan includes KMC:** Costed national implementation plans for maternal newborn health include KMC	**Yes** = costed plan or plans to scale up maternal, newborn and child health intervention includes KMC components<br>**No** = no costed implementation plan OR costed implementation plan does not include KMC components	Costed plans - record review; Key informants through interview	Indicator does not evaluate whether the costed plan is adequate to cover the implementation of KMC in line with global recommendations. Costed plans may not reflect actual expenditure and may be updated infrequently.	Documents should be provided to substantiate a 'yes' value. Special costing analysis may be required on periodic basis to assess how well KMC is budgeted for.
**KMC service availability:** Percentage of facilities with in-patient maternity services with operational KMC	<u>Numerator</u>: Number of health facilities in which KMC is operational [(1)]<br><u>Denominator</u>: Number of health facilities with inpatient maternity services	Facility assessments and MOH records (collected through supervision or periodic audits)	Consensus on definition of 'operational' KMC is lacking and there are gaps in what current health facility assessment tools capture. Facility assessments can be expensive to conduct regularly and only capture a single point in time and often only cover a sample of facilities.	Countries can develop tailored definitions/criteria for 'operational' KMC that best reflect their protocols. In some settings, it may be possible to develop a system to track facilities offering KMC and update annually based on country-defined criteria.
**Weighed at birth:** Percentage of newborns weighed at birth	<u>Numerator</u>: Number of newborns weighed at birth[(2)]<br><u>Denominator:</u> Number of live births	Interviews with mothers + child health card review - collected through household surveys; L&D registers - collected through record review as part of facility assessment or supervision	Maternal recall of child weighing may be biased and accuracy of child health cards is unknown and likely to vary greatly; household surveys are only conducted periodically and my not provide sub-national estimates. Birthweight may not be recorded in L&D registers or may be just estimated in the absence of scales.	See notes related to timeframe[(2).] Data quality audits of L&D records could be conducted to assess quality of data on birthweight at health facilities and on child health cards and used to identify the most reliable and feasible approach to data collection. Facilities should be encouraged to track their performance on this indicator as a basic element of newborn care.

Indicator	Metric	Data source(s) and methods of collection	Limitations/challenges	Data collection considerations and possible alternatives
**Identification of newborns ≤2000g:** Percentage of live births identified as ≤2000g	Numerator: Number of newborns identified as ≤2000g<br>Denominator: Number of live births	L&D registers - collected through HMIS (see notes) or through register review as part of supervision or facility assessment	Most facilities track and report on the number of babies <2,500g (definition of LBW) and data on babies ≤2000g would need to be extracted from register reviews. Quality of birthweight data in L&D registers may be poor and record reviews would often only capture single point in time.	This is the ideal denominator for assessing coverage of KMC and countries should work towards developing systems to capture this information over time. As noted for the indicator above, data quality audits are recommended to assess current status of L&D data on birthweight and facilities should be encouraged to collect and report on this information to assess coverage of KMC in their own facilities.
**KMC coverage*:** Percentage of newborns initiated on facility-based KMC	Numerator: Number of newborns initiated on facility-based KMC[3]<br>Denominator: Expected number of live births OR expected number of LBW babies	KMC registers - reported through HMIS or collected through register review as part of facility assessment; Denominator available through national and global estimates updated annually	Indicator does not capture quality of KMC services or adherence to recommended practices. The ideal denominator would be number of babies ≤2000g, but as noted, this is not typically available, and alternative denominators are more challenging to interpret (see note 3).	KMC initiation needs to be defined at country level so that a standard approach is used for tracking. Work is underway through ENAP metrics to improve denominator estimates and establish benchmarks for interpretation (see note 3).
**KMC monitoring:** Percentage of KMC newborns who are monitored by health facility staff according to protocol	Numerator: Number of newborns admitted to KMC who are monitored by health facility staff according to protocol (includes at minimum: assessing feeding, STS duration, weight, temperature, breathing, heart rate, urine/ stools)<br>Denominator: Number of newborns initiated on facility-based KMC	KMC patient charts - collected through record review as part of facility assessment/supervision visits	Indicator does not capture whether KMC is provided according to protocol. Patient charts may not capture the required information or may be poorly filled.	Countries need to tailor the definition to their own protocol and patient charts. Assessment of this information for the complete duration of stay in facility-based KMC may be difficult and an alternative option would be to select a timeframe and review completeness of KMC monitoring for all babies in KMC during that time period. Facilities should be encouraged to track their performance on this indicator and establish targets.

Indicator	Metric	Data source(s) and methods of collection	Limitations/challenges	Data collection considerations and possible alternatives
**Status at discharge from KMC facility:** Percentage of newborns discharged from KMC facility who: met facility criteria for weight gain/health status; left against medical advice; referred out; or died before discharge	Numerator: Number of newborns discharged from facility-based KMC who: 1) met facility criteria for weight gain, health status, feeding, thermal regulation, family competency, etc; 2) left against medical advice; 3) referred out for higher level care; 4) died before discharge<br>Denominator: Number of newborns discharged from facility-based KMC	KMC registers - reported through HMIS or collected through register review as part of facility assessment	Indicator can be challenging to interpret and compare across facilities in the absence of global standards (as exist for management of acute malnutrition programs for example) and would need to be disaggregated by level of care and birthweight.	Countries need to develop clear definitions for each category of status at the time of discharge and ensure that facility records capture these. Facilities should be encouraged to track their performance on this indicator and establish targets (ideally by birthweight category).
**KMC follow-up:** Percentage of newborns discharged from facility-based KMC that received follow-up per protocol	Numerator: Number of newborns discharged from facility-based KMC that received follow-up per protocol<br>Denominator: Number of newborns discharged alive who received facility-based KMC(4)	KMC registers/records – reported through HMIS or collected through register review as part of facility assessment and/or) Interviews with caregivers/mothers of newborns discharged from KMC	Indicator does not capture quality of follow-up care or outcomes of babies discharged from facility-based KMC. Refer to note 4 regarding the denominator. Tracking follow-up at individual facility level can be challenging in cases where follow-up is scheduled at a different facility.	Countries will need to define what constitutes follow-up care according to protocol and what is feasible to monitor. Ideally completion of follow-up care could be tracked and reported on routinely; however in settings accurate capture may be too difficult and periodic assessments may be a better alternative.

Notes:

(1) KMC elements already collected through Service Provision Assessments (SPA) include: staff receiving in-service training on KMC; identified space for KMC; and availability of functional infant scale. This indicator has been prioritized for further testing by the KMC Acceleration and ENAP metrics group, with particular focus on identifying and testing additional KMC elements for inclusion in future harmonized facility assessments, supervision checklists and MOH audits.

(2) Countries will need to define a timeframe for 'weighed at birth'. In some settings, this may include babies weighed at admission to the health care facility within a certain timeframe after delivery and babies weighed at home by a trained provider with weight documented on a maternal held record.

(3) This may include facility-initiated ambulatory KMC as in Latin America (e.g. Colombia); this indicator has been prioritized for further testing by the KMC Acceleration and ENAP metrics group, with particular focus on establishing the most feasible, valid, and reliable denominator and benchmarks for interpretation.

(4) Countries should define their own denominator based on the national protocol for follow-up care of small and sick newborns, with an ideal denominator that captures all those infants discharged alive that were potential candidates for KMC

* - Indicator recommended as priority for inclusion in national HMIS

Acronyms: KMC = Kangaroo Mother Care; HMIS = Health Management Information System; MOH = Ministry of Health; LBW = low birth weight; L&D = Labour and Delivery; STS = skin-to-skin;
